# Two cases of intrahepatic cholangiocellular carcinoma with high insertion-deletion ratios that achieved a complete response following chemotherapy combined with PD-1 blockade

**DOI:** 10.1186/s40425-019-0596-y

**Published:** 2019-05-07

**Authors:** Minghao Sui, Yu Li, Hongguang Wang, Ying Luo, Tao Wan, Xun Wang, Bingyang Hu, Yanshuang Cheng, Xianrong Lv, Xianlei Xin, Qiang Xu, Guan Wang, Shichun Lu

**Affiliations:** 1 Department of Hepatobiliary Surgery, the First Medical Center, Chinese PLA General Hospital, Medical School of Chinese PLA, 28 Fuxing Road, Beijing, 100853 China; 2 Radiotherapy Department, Chinese PLA General Hospital, Beijing, 100853 China; 3 GenomiCare Biotechnology (Shanghai) Co., Ltd., Shanghai, 201210 China

**Keywords:** Indel, Intrahepatic cholangiocarcinoma, Combination immunotherapy, PD-1 blockade, Whole-exome sequencing

## Abstract

**Background:**

Insertion–deletion mutations (indels) may generate more tumour-specific neoantigens with high affinity to major histocompatibility complex class I. A high indel ratio is also related to a good response to programmed death-1 (PD-1) checkpoint blockade in melanoma and renal cell carcinoma. However, the correlation between a high indel ratio and the immunotherapy response in intrahepatic cholangiocarcinoma (ICC) is unknown.

**Case presentation:**

Two patients with relapsed ICC at stage IIIb were treated with PD-1 blockade combined with chemotherapy. After 7 and 4 months of chemotherapy and PD-1 blockade (3 and 15 cycles, and 5 and 6 cycles, respectively), magnetic resonance imaging and positron emission tomography with computed tomography imaging showed that both patients achieved a complete response (CR), which has lasted up to nearly 16 and 13 months to date, respectively. Whole-exome sequencing and immunohistochemistry analysis showed that both patients had cancers with microsatellite stability (MSS) and mismatch repair (MMR) proficiency, weak PD-L1 expression, and a tumour mutation burden (TMB) of 2.95 and 7.09 mutations/Mb, respectively. Patient 2 had mutations of *TP53* and *PTEN* that are known to confer sensitivity to immunotherapy, and the immunotherapy-resistant mutation *JAK2*, whereas patient 1 had no known immunotherapy response-related mutations. However, the indel ratios of the two patients (48 and 66.87%) were higher than the median of 12.77% determined in a study of 71 ICC patients. Moreover, comparison to six additional ICC patients who showed a partial response, stable disease, or progressive disease after PD-1 blockade treatment alone or in combination with chemotherapy demonstrated no difference in PD-L1 expression, TMB, MSI, and MMR status from those of the two CR patients, whereas the indel frequency was significantly higher in the CR patients.

**Conclusions:**

These two cases suggest that indels might be a new predictor of PD-1 blockade response for ICC patients beside PD-L1 expression, TMB, MSI, and dMMR, warranting further clinical investigation.

**Electronic supplementary material:**

The online version of this article (10.1186/s40425-019-0596-y) contains supplementary material, which is available to authorized users.

## Background

Intrahepatic cholangiocarcinoma (ICC) is an aggressive malignancy with a poor prognosis. After curative resection, the 5-year survival rate and median survival time of ICC patients is 30% and 28 months, respectively [1], and the recurrence rate has been reported to be in the range of 40–80% [2]. Other than surgical resection, standard treatment options for ICC include liver transplantation, a gemcitabine-based chemotherapeutic regimen, and loco-regional therapies such as transarterial chemoembolization and conformal external-beam radiation therapy; however, given the poor outcome, more effective treatments are urgently needed.

Similar to virus-associated cancers, viral hepatitis and *Clonorchis sinensis* infection are known risk factors for ICC [3]. These infections often cause immune exhaustion, which is mediated through the programmed cell death 1-ligand 1 (PD-L1)/programmed cell death 1 (PD-1) pathway, similar to the immunosuppressive mechanism of cancer [4]. Indeed, several studies have shown that PD1/PD-L1 blockade can be effective in the treatment of virus-related cancers [5]. Moreover, a study including 27 patients with ICC showed that 100, 30, and 41% of the cases had infiltrated lymphocytes, positive PD-L1 expression, and positive human leukocyte antigen class I antigen (HLA I) expression, respectively [6]. In another study, 39 of 54 patients with ICC were found to be positive for PD-L1 expression within the tumour front using immunohistochemistry (IHC), and the overall survival of these patients was reduced by approximately 60% compared with that of patients without PD-L1 expression [7]. Similarly, 260 patients with biliary tract cancer (BTC) that had a relatively poor prognosis showed higher PD-L1 expression [8]. Collectively, these studies provide a biological rationale for the treatment of ICC patients with PD1/PD-L1 blockade.

More importantly, clinical trials have demonstrated a benefit of PD-1/PD-L1 blockers for patients with ICC. The PD-L1 positive BTC cohort of the KEYNOTE-028 basket trial showed that four of 24 patients who were PD-L1 positive showed a partial response (PR), and four patients had stable disease (SD) [9]. Another basket trial including four patients with DNA mismatch repair deficiency (dMMR) cholangiocarcinoma demonstrated that one patient had a complete response (CR) and the other three patients had SD after PD-1 blockade therapy [10]. Further, three recent studies reported encouraging clinical outcomes. In a Phase II study on patients with advanced refractory BTC treated with nivolumab, 17% of the 29 patients achieved a PR, 38% showed SD, and there was an overall 55% disease control rate (DCR). No grade IV or V toxicities were reported [11]. Asian BTC patients that received M7824 after chemotherapy failure, which targets PD-1 and transforming growth factor-β, showed durable responses with a 40% objective response rate for ICC [12]. However, these studies did not determine the molecular characteristics of the ICC patients that showed a clinical benefit from the treatment. Furthermore, the combination of pembrolizumab and chemotherapy has also shown a good response in a case report of a single ICC patient. This ICC patient had a high tumour mutation burden (TMB) of 19.3 mutations/Mb, but with microsatellite instability (MSI) and MMR proficiency (pMMR) [13]. Thus, identifying the subsets of ICC patients that are most likely to benefit from PD1/PD-L1 blockade alone and in combination remains a challenge and hindrance to effective personalized medicine.

Insertion–deletion mutations (indels) cause frameshift mutations that not only alter the amino acid composition of the proteins but may also lead to early termination of protein synthesis. Indels and single-nucleotide variants (SNVs) together determine the TMB. Notably, indels could produce more than three times the neoantigens with high affinity to major histocompatibility complex class I (MHC-I) (IC50 < 50 nM), and nine times the mutation-specific neoantigens compared with SNVs. This high neoantigen load induced by indels was associated with HLA I presentation, CD8^+^ T cell activation, and increased cytolytic activity compared with the high SNV neoantigen group [14]. Consequently, the indel count has been significantly associated with a response to a checkpoint inhibitor across three separate melanoma cohorts and in patients with advanced renal cell carcinoma [14, 15]. However, there are limited data on the ability of indels to predict a response to PD-1/PD-L1 blockers alone and in combination. Here, we report two patients with ICC with high indel ratios who were successfully treated with PD-1 blockers plus chemotherapy, both of whom showed weak PD-L1 expression and a microsatellite stable (MSS) status, and without dMMR. Moreover, one of the patients had a relatively low TMB. Comparison of the features of these patients to others reported in the literature, as well as to six additional cases of ICC that received PD-L1 therapy without a CR, points to a potential role of indels as a key factor determining the response to therapy, warranting further consideration.

## Case presentation A (Patient 1)

**Fig. 1:**
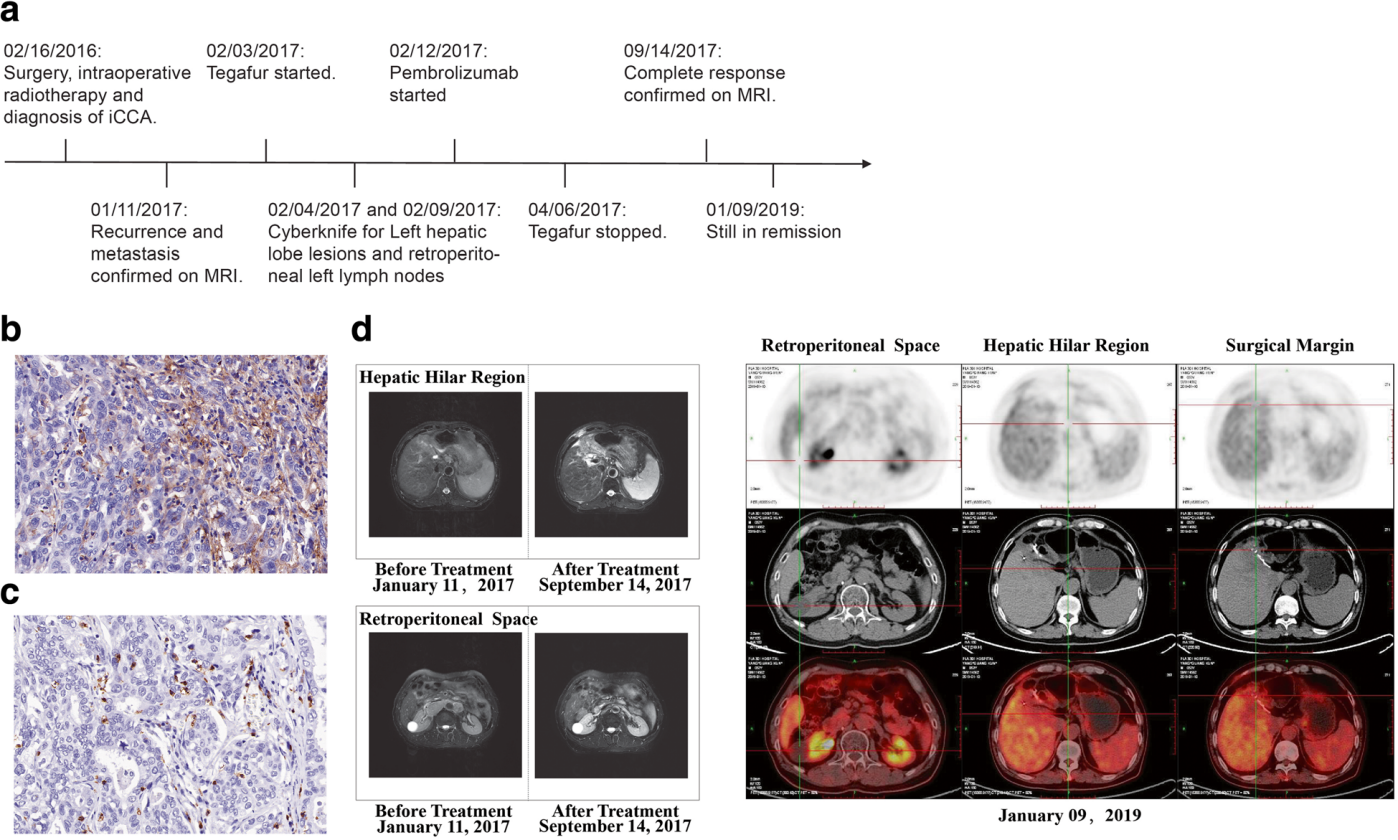
Imaging of patient 1 over the course of therapy. **a** showed timeline of the clinical course. Representative micrographs of PD-L1 expression (**b**) and percentage of CD8^+^ T cells (**c**) within tumor (original magnification ×400). The positive rate of PD-L1 and CD8^+^ T cells were < 5% and 10%, respectively. MRI and PET-CT imaging (**d**) showed the lesion around the margin and enlarged lymph nodes disappeared after treatment with pembrolizumab in combination with tegafur on September 14, 2017. The CR was sustained up to 9 January 2019

Patient 1 (Fig. 1 and Table 1) is a 50-year-old male with moderately differentiated ICC staged at IIIb. He was admitted to the hospital in January 2016 due to upper abdominal pain. He had a history of hepatitis B for 10 years, and his Child-Pugh class was A. Magnetic resonance imaging (MRI) revealed a mass in the left outer lobe, which grew outward and invaded the diaphragm. The tumour marker carcinoembryonic antigen was elevated at 10.14 μg/L. He underwent left hemihepatectomy and hepatoduodenal ligament skeletonization on February 16, 2016. The tumour was 11 cm × 9 cm × 6 cm, and no lymph node metastases were found. Intraoperative radiotherapy was performed on the liver section using 9-mV photon beams with a single dose of 12 Gy, which could eliminate the residual tumour due to invasion of the diaphragm and the venous root of the liver. The tumour was found to be positive for cytokeratin 18 (CK18) and was negative for Arg-1, hepatocyte, glypican-3 (GPC-3), and CK7 in IHC analysis. The tumour proportion score (TPS) of the PD-L1 expression level was < 5% determined using monoclonal mouse anti-human PD-L1 clone (22C3) antibody by allred criteria, and the frequency of infiltrating CD8^+^ T cells was 10%.

**Table 1:** Baseline, mutation characteristics, treatment, and outcomes

	Patient								Reference
	1	2	3	4	5	6	7	8	
*Baseline characteristics*									
Age (years)	50	67	60	51	65	59	55	60	
Sex	male	male	female	female	female	male	male	male	
Surgery	Yes	Yes	Yes	No	Yes	Yes	No	Yes	
Stage	IIIb	IIIb	IIIb	IIIb	IIIb	IIIb	IV	IIIb	
Lymph node metastasis	+	–	+	+	+	+	+	+	
Virus infection	HBV	NHVI	NHVI	NHVI	NHVI	NHVI	HCV	HBV	
Tumour PD-L1 expression level (%)	< 5%	< 5%	< 1%	unknown	> 90%	> 90%	unknown	< 5%	
Percentage of CD8^+^ T cells	10%	10%	10%	unknown	20%	20%	unknown	30%	
*Mutation characteristics*									
TMB (mutations/Mb)	2.95	7.09	5.62	4.18	8.47	34.84	5.38	6.01	7.4 (median) [21]
TMB (NSM)	25	163	114	66	209	1088	106	127	47 (median) [22]
Number of SNVs	13	54	85	52	173	771	82	105	41 (median) [22]
Number of indels	12	109	29	14	36	317	24	22	6 (median) [22]
indels ratio (%)	48.00%	66.87%	25.44%	21.21%	17.22%	29.16%	22.64%	17.32%	12.77% (median) [22]
MSI-H/MSS	MSS	MSS	MSS	MSS	MSS	MSI-H	MSS	MSS	3.2% MSI-H [20]
dMMR/pMMR	pMMR	pMMR	pMMR	pMMR	pMMR	dMMR	pMMR	pMMR	2% dMMR [10]
Percentage of CNVs (%)	3.1	4.2	9.29	2.66	4.75	0.78	3.67	5.92	
Mutations conferring sensitivity to immunotherapy	NA	*TP53/PTEN*	*KRAS*	NA	*BRAF/GITR*	*MLH1/GITR*	*TP53*	*TP53*	
Mutations conferring resistance to immunotherapy		*JAK2*	*B2M*				chromosome 11q13		
*Treatment*									
Therapeutic regimen (cycles)	tegafur (3) + pembrolizumab (15) + cyberknife (2)	tegafur (5, but irregular) + pembrolizumab (6)	nivolumab (4) + cisplatin (1) + gemcitabine (1)	nivolumab (15)	nivolumab (8)	nivolumab (8) + cisplatin (1) + gemcitabine (1)	nivolumab (8) + lenvatinib (8 months continuously)	pembrolizumab (6)	
*Outcome*									
Type of response	CR	CR	PD	SD	PD	PR	PD	PR	
PFS (months)	16	13	Unknown	7	5	3	Unknown	10	

The whole exons of tumour tissues removed during surgery and matched white blood cells were sequenced by the Illumina NovaSeq 6000 Sequencing System with average sequence coverage of 200X. We calculated SNVs, indels, and copy number variations (CNVs) by the mutect2 algorithm, and CNVnator software, respectively. MSI was identified using msisensor, and dMMR was identified by analysing SNVs and indels in *MLH1, MSH2, MSH6*, and *PMS2*

*Abbreviations*: *CNG* copy number gain, *CNL* copy number loss, *NHVI* no hepatitis virus infection

Liver resection margin recurrence and abdominal lymph node metastasis were detected using MRI and positron emission tomography-computed tomography (PET-CT) after 11 months. MRI showed a marginal lesion of 4 × 1.5 cm in the left lobe of the liver, along with an enlarged hepatic hilar (1.6 × 1.5 cm) and retroperitoneal lymph nodes (5.2 × 3 cm and 2.8 × 2.6 cm). PET-CT scans also revealed abnormal hypermetabolic lesions in these locations.

Whole-exome sequencing (WES) was applied to the tissue resected after surgery, and the data were used to determine the presence of SNVs, indels, the TMB, copy number variations (CNVs), MSI status, and dMMR by bioinformatics methods. The TMB was determined to be 2.95 mutations/Mb, and a total of 25 non-synonymous mutations (NSMs) were detected in the whole genome, including 12 indels and 13 SNVs. This patient harboured only one clinically actionable mutation in *FGF4*, which amplified to reveal a copy number of 3.64. No SNVs were detected in *MLH1, MSH2, MSH6*, and *PMS2* (Additional file 1), suggesting pMMR, and the MSI was 0.01%.

The left hepatic lobe lesions and retroperitoneal left lymph nodes were treated with Cyberknife (52 Gy/4 F for 4 days) on February 4, 2017 and February 9, 2017, respectively. Tegafur and pembrolizumab were initiated 9 days apart. Tegafur chemotherapy was started at 40 mg twice a day every 3 weeks but was withdrawn due to development of thrombocytopenia and leukopenia after three cycles. Pembrolizumab was administered at 150 mg every 3 weeks for 15 cycles for about 1 year. On June 6, 2017, the abdominal MRI showed that most of the lesions in the margin, and all the lesions in the hilar and retroperitoneal lymph nodes had disappeared. It was speculated that the residual lesions at the margin might represent a surgical reaction. The patient was deemed to show a CR on September 14, 2017 and was still in remission at the last follow-up of January 10, 2019 based on PET-CT analysis. No side effects of pembrolizumab were observed.

## Case presentation B (Patient 2)

**Fig. 2:**
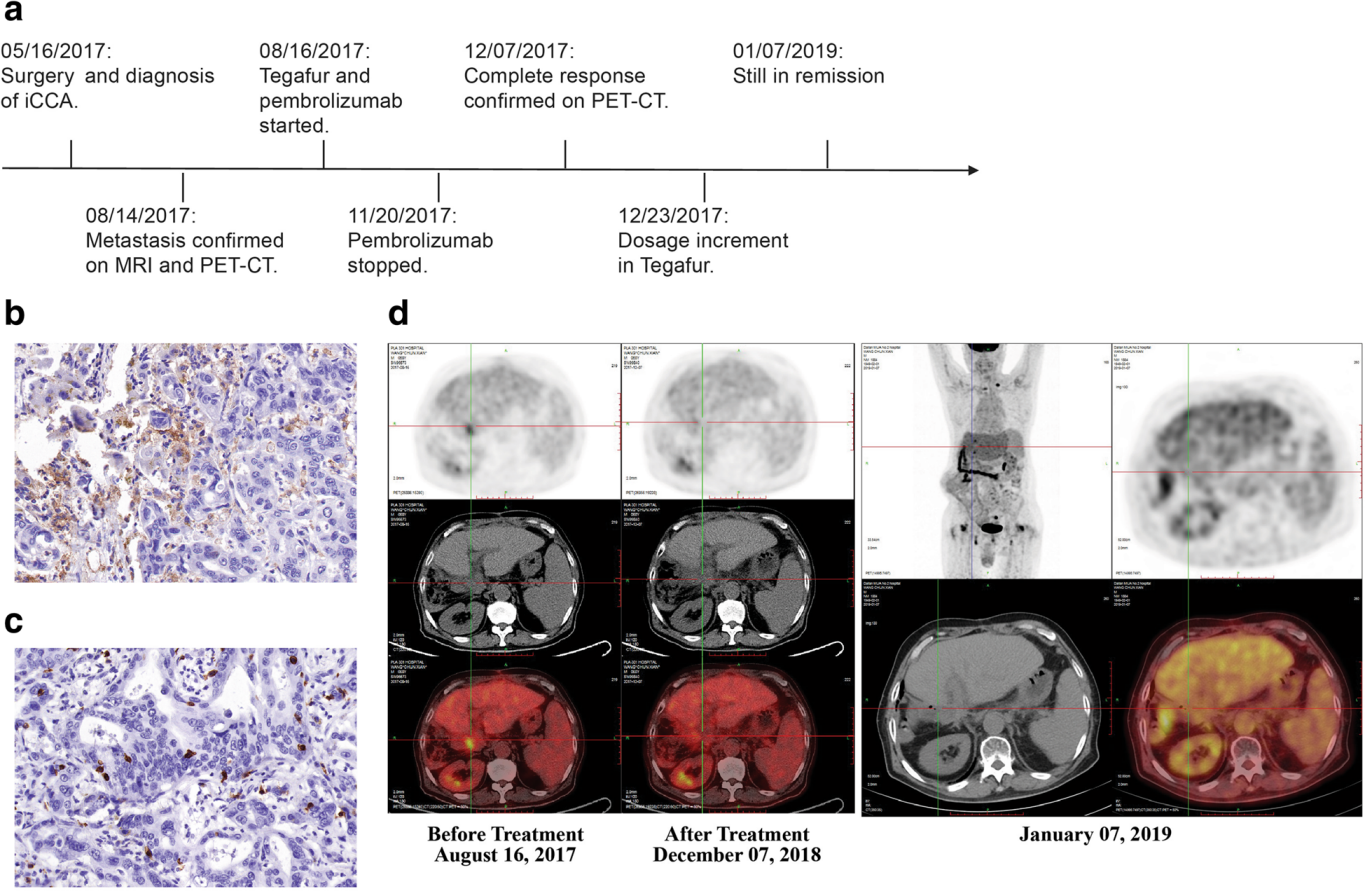
**a** Timeline of the clinical course in patient 2. The positive rate of PD-L1 (**b**) and CD8^+^ T cells (**c**) in patient 2 were < 5% and 10% evaluated by IHC, respectively (magnification ×400). Patient 2 had a complete metabolic response after treatment with pembrolizumab in combination with tegafur, with no residual hypermetabolic uptake on post-treatment imaging (**d**)

Patient 2 (Fig. 2 and Table 1) is a 67-year-old male with no hepatitis virus infection. He underwent extended right hemihepatectomy, left hepaticojejunostomy, perihepatic lymphadenectomy, and portal vein reconstruction on May 16, 2017. The tumour measured 7.6 cm × 7 cm × 7 cm, with nerve invasion accompanied by microvascular invasion. No tumour was found in the liver margin and bile duct margin after the surgery. Lymphatic metastasis was detected in groups 8 and 12A. IHC showed Arg-1 (−), CK18 (+), GPC-3 (−), hepatocyte (−), Ki-67 positivity of 65%, and CK19 (+). Accordingly, he was diagnosed with iCCA stage IIIb. Similar to Patient 1, PD-L1 expression was detected on < 5% of the tumour cells, and the percentage of CD8^+^ T cells was 10%.

Lymph node metastasis in the hepatoportal area was detected using MRI and PET-CT on August 16, 2017. WES revealed 163 NSMs, including clinically actionable alterations in *PTEN* and *TP53*. In addition, *TP53, SMAD4*, and *ARID2* are included in the COSMIC top 20 mutated genes (Additional file 1). The TMB was 7.09 mutations/Mb, including 109 indels (66.87%) and 54 SNVs. The tumour exhibited pMMR and MSI (0.01%). He was started on a regimen of tegafur and pembrolizumab in late August. Unfortunately, he experienced the common adverse event to tegafur of pruritus, determined to be of grade 2 according to the standard CTCAE5.0 criteria. After withdrawing tegafur, the pruritus disappeared, and the drug was thus switched to an irregular administration schedule as of December 23, 2017 with an increase in the dose from 40 mg to 60 mg and to be taken twice a day until the beginning of February 2018. Pembrolizumab was administrated intravenously (150 mg every 3 weeks for six cycles). PET-CT scans revealed that the enlarged lymph nodes had disappeared. This CR was achieved in less than 4 months, and the patient continued to be in remission for 13 months up to the last follow-up.

## Discussion

This is the first report demonstrating two ICC patients with high indel rates that achieved a CR after PD-1 blockade combined with chemotherapy. We also analysed the presence of genomic alterations known to be involved in the response and resistance to immunotherapy using WES [16–18]. Patient 2 harboured three such mutations, including *TP53* and *PTEN* inactivating mutants, which can induce a strong potential response, and *JAK2* mutations, which have been associated with immune therapy resistance. However, Patient 1 did not have any related mutations. Both patients had an MSI frequency of 0.01%, which is well within the spectrum of MSS tumours [19, 20], and also showed a pMMR molecular phenotype.

The median TMB of patients with ICC varies among studies. However, the TMB levels of Patients 1 and 2 were lower or equal to the median reported for 69 Chinese ICC patients [21]. In addition, the TMB of Patient 1 was half the median TMB reported for 71 cholangiocarcinoma patients, whereas that of Patient 2 was nearly four times higher than the median [22]. Moreover, both patients of this study showed weak PD-L1 expression (< 5% of tumour cells), suggesting a weak PD-L1 status. However, IHC showed that CD8^+^ T cell infiltration was moderate at 10% in both patients. A previous report showed that patients with a higher density of CD8^+^ T cells at the edge of tumour invasion respond better to treatment [23]; thus, infiltrated CD8^+^ T cells may be a prerequisite for effective immunotherapy. Overall, although these two patients did not show typical characteristics of an immunological benefit such as a high TMB, high MSI, and dMMR, compared with the median indel rate of 12.77% for ICC patients [22], the indel rates of these two patients were substantially higher at 48 and 66.87%, respectively. Moreover, the CR lasted for 16 and 13 months, respectively, as of January 2019, consistent with previous reports that high indel counts are associated with a good response to immunotherapy in melanoma and renal cell carcinoma [14, 15].

For comparison, we also analysed the cases of an additional six ICC patients that received PD-1 treatment, alone or in combination, but did not show a CR (Table 1). Patients 4, 5, and 8 were treated with PD-1 blockade alone; patients 3 and 6 received a combination of PD-1 blockade and chemotherapy; and patient 7 was treated with a combination of PD-1 blockade and lenvatinib. Among the patients showing a PR, patient 6 had high-MSI status and dMMR, and also showed the highest TMB of 34.84 mutations/Mb with high PD-L1 expression (> 90%). By contrast, patient 8 also showed a PR and did not share these characteristics, demonstrating weak PD-L1 expression (< 5%). Among the patients that showed progressive disease (PD), patient 5 had high PD-L1 (> 90%) and an inactivated *BRAF* mutation, which is associated with sensitivity to immunotherapy. Patients 3 and 7 harboured both immunotherapy-sensitive and -resistant mutations, demonstrating similar complexity in the mutation profile as determined for Patient 2 (Additional file 1). However, these mutations were only analysed at the DNA levels, and thus transcriptome analysis is necessary for confirmation. Compared with the PR, SD, and PD groups of these six additional patients, Patient 1 had the lowest TMB and Patient 2 had a TMB that was not significantly different from that of the other patients. However, the indel rate of the CR groups was higher than that of the other groups, and was significantly different from that reported previously [22] (*P* = 0.001 and *P* < 0.001, respectively).

Immunotherapy has achieved great success, with 10–35% of patients showing a response to single immune checkpoint blockade inhibitors [24]. Recently, more clinical effort has focused on combination immunotherapy such as immune checkpoint inhibitors combined with chemotherapy, radiotherapy, and/or targeted therapy. Chemotherapy can increase the immunogenicity of tumour cells mainly by regulating the antigenicity and adjuvanticity [24]. Chemotherapy drugs can destroy genes and trigger new mutations, thereby increasing antigenicity, although these new antigens appear to be expressed at lower levels in tumours [25]. Chemical agents have been developed that trigger immunogenic cell death, resulting in the release of damage-associated molecular patterns to subsequently activate inherent and adaptive immune responses. Chemotherapy also depletes regulatory T cells and myeloid-derived suppressor cells, which may result in the formation of further hot immune tumours [26].

Trials of the combination of chemotherapy with an immune checkpoint blocker (CIT) have been completed for patients with non-small cell lung cancer (NSCLC), demonstrating that CIT was more effective than chemotherapy alone. In two phase-3 studies (KEYNOTE-189 and KEYNOTE-407), pembrolizumab plus pemetrexed and platinum-based or carboplatin and taxane chemotherapy both showed a significantly improved response rate (47.6% vs. 18.9% and 58.4 vs. 35.0%) and progression-free survival (PFS) (8.8 months vs. 4.9 months and 6.4 months vs. 4.8 months, respectively). Nivolumab and atezolizumab combined with chemotherapy also showed positive results. In the PACIFIC trial, patients with locally advanced unresectable NSCLC received chemoradiation followed by durvalumab, which resulted in significant improvement in median PFS and 18-month PFS rates compared chemoradiation followed by placebo [27]. Moreover, among 14 patients with ICC who failed previous anticancer therapy that received lenvatinib combined with pembrolizumab or nivolumab, three patients achieved a PR and the DCR was 92.9%. A total of 450 cancer genes and whole exons were sequenced in seven patients, revealing four patients with a high TMB greater than 12 mutations/Mb, and one of the of four was MSI-H [28].

In the KEYNOTE-189 and KEYNOTE-407 trials, patients with less than 1% PD-L1 expression also responded to CIT, and there was no association of PD-L1 expression with a clinical benefit [27]. The expression of PD-L1 may be affected by both time fluctuations and intratumoural heterogeneity, so that it is not always associated with a better outcome. Moreover, one ICC patient with a low TMB showed tumour shrinkage on a regimen of lenvatinib combined with PD-1 blockade [28]. A study on pancreatic cancer suggested that the quality of a tumour antigen could better predict the survival of patients after surgery but not the number of tumour antigens [29]. Our present cases also showed that a high TMB, MSI-H, and PD-L1 expression cannot completely predict all patients who will benefit from combination immunotherapy. Hence, it is still an unmet need to identify which patients may receive the benefit from single or combined immunotherapy.

Compared with immunotherapy alone, it is more difficult to explore how the combination of conventional therapy might promote immunotherapy and to screen patients that will receive clinical benefits, as biopsy sampling is required, which is not standard in routine clinical practice. In addition to PD-L1 expression, high MSI, dMMR, and high TMB which have been approved by the Food and Drug Administration as biomarkers of immunotherapy [30], our results further suggest that the indel ratio may be associated with a response to PD-1 blockade for ICC patients. However, clinical studies with larger samples are required to validate this association and understand the underlying mechanism.

## Additional file

Additional file 1: List of mutated genes in the eight ICC patients, and comparison with the top 20 COSMIC mutated genes, and the list of sensitive or resistant mutations to immunotherapy. (XLSX 12 kb)

## Abbreviations

BTC: Biliary tract cancer
CIT: Combination of chemotherapy with an immune checkpoint blocker
CNVs: Copy number variations
CR: Complete response
DCR: Disease control rate
dMMR: DNA mismatch repair deficiency
FDA: Food and Drug Administration
ICC: Intrahepatic cholangiocarcinoma
IHC: Immunohistochemistry
Indels: Insertion–deletion mutations
MHC-I: Major histocompatibility complex class I
MRI: Magnetic resonance imaging
MSI-H: High microsatellite instability
MSS: Microsatellite stable
NSCLC: Non-small cell lung cancer
NSMs: Nonsynonymous mutations
PD-1: Programmed cell death protein 1
PD-L1: Programmed cell death 1-ligand 1
PET–CT: positron emission tomography with computed tomography
pMMR: MMR proficiency
PR: Partial response
SD: Stable disease
SNVs: Single-nucleotide variants
TMB: High tumour mutation burden
WES: Whole-exome sequencing
